# Pregnancy-Induced Changes in microRNA Expression in Multiple Sclerosis

**DOI:** 10.3389/fimmu.2020.552101

**Published:** 2021-01-28

**Authors:** Helle Bach Søndergaard, Laura Airas, Jeppe Romme Christensen, Birgitte Romme Nielsen, Lars Börnsen, Annette Oturai, Finn Sellebjerg

**Affiliations:** ^1^ Danish Multiple Sclerosis Center, Copenhagen University Hospital, Rigshospitalet, Glostrup, Denmark; ^2^ Department of Neurology, Turku University Hospital, Turku, Finland

**Keywords:** multiple sclerosis, pregnancy, third trimester, postpartum, microRNA

## Abstract

Pregnancy affects the disease course in multiple sclerosis (MS), particularly in the third trimester, where the relapse rate is reduced by as much as two thirds. This study aimed at identifying changes in microRNA (miRNA) and immune cell phenotypes in pregnant MS patients. Discovery and validation studies to detect differentially expressed miRNAs were performed with quantitative real-time PCR on peripheral blood mononuclear cells (PBMC). Flow cytometry analysis was performed on PBMC stained with antibodies directed against surface markers of antigen presenting cells (APCs), NK-cells, NKT cells, CD4+ and CD8+ T cells and subsets of these cell types, including PDL1 and PDL2 expressing subsets. RNA was extracted from whole blood, monocytes, and NK-cells to investigate expression and correlation between regulated miRNAs and mRNAs. In total, 15 miRNAs were validated to be differentially expressed between third trimester pregnant and postpartum MS patients (Benjamini-Hochberg false discovery rate from p = 0.03–0.00004). Of these, 12 miRNAs were downregulated in pregnancy and 6 of the 15 miRNAs were altered by more than ±2-fold (+2.99- to -6.38-fold). Pregnant MS patients had a highly significant increase in the percentage of monocytes and a decrease of NK-cells and myeloid dendritic cells compared to non-pregnant MS patients. We confirm previous reports of a relative increase in CD56-bright NK-cells and a decrease in CD56-dim NK-cells in third trimester of pregnancy and report an increase in non-committed follicular helper cells. *PDL1* and *PDL2* expression was increased in pregnant patients together with *IL10*. Also, in monocytes *IL10*, *PDL1*, and *PDL2* were upregulated whereas miR-1, miR-20a, miR-28, miR-95, miR-146a, miR-335, and miR-625 were downregulated between pregnant and untreated MS patients. *IL10*, *PDL1*, and *PDL2* were predicted targets of MS pregnancy-changed miRNAs, further supported by their negative correlations. Additionally, previously identified pregnancy-regulated mRNAs were identified as predicted targets of the miRNAs. PDL1 and PDL2 bind PD-1 expressed on T cells with an inhibitory effect on T-cell proliferation and increase in IL10 production. These results indicate that some of the effects behind the disease-ameliorating third trimester of pregnancy might be caused by changed expression of miRNAs and immunoregulatory molecules in monocytes.

## Introduction

Pregnancy affects the disease course in relapsing-remitting multiple sclerosis (RRMS), particularly in the 3^rd^ trimester where the relapse rate is reduced by up to 70% (1, 2). This is followed by an increased relapse rate in the first months postpartum, coinciding with the rapid decrease in pregnancy hormone levels. A similar effect is also observed for other autoimmune diseases such as rheumatoid arthritis (RA) and uveitis (3). The reduction in MS disease activity during pregnancy is thought to be caused by a shift towards a more immune tolerant and anti-inflammatory state which allows the immune interactions at the feto-maternal interphase needed for a successful pregnancy (4).

The molecular mechanisms behind these changes are incompletely understood, but previously pregnant MS patients were shown to have an overall increase of the regulatory CD56^bright^ natural killer (NK)-cells and a decrease in CD56^dim^ NK–cells (2). CD56^dim^ cells have been associated with disease activity (5) and a decrease in the percentage of CD56^bright^ together with an increase in CD56^dim^ NK-cells was reported to correlate with MRI activity in MS (6). Pregnancy-induced changes in mRNA expression have furthermore been studied in the 3^rd^ trimester compared to the postpartum period in MS patients and healthy controls, identifying eleven non-HLA genes differentially expressed in MS patients in one study (7) and a signature of seven genes known to be inflammatory regulators in another study (8). A follow-up study of these signature genes also identified a significantly lower gene expression level of *SOCS2, NR4A2*, and *TNFAIP3* in MS patients with an aggressive disease course that correlated with expanded disability status scale (EDSS) change and relapse rate (9).

Epigenetic regulation by microRNAs (miRNAs) influences a wide range of physiological and pathological processes by their binding to mRNA targets through antisense interactions (10). In mammals two different miRNA repression mechanisms occur where the dominating is mRNA destabilization (66–90%) followed by translational repression (10). Thus, immune cell subsets and mRNA expression during pregnancy might be influenced by changes in miRNAs by their addition of an extra layer of post-transcriptional gene control.

The increase in steroids with anti-inflammatory or neuroprotective effects, such as estriol, estradiol, progesterone, cortisol, and preimplantation factor might induce disease amelioration in MS patients during pregnancy. These steroids have been investigated in mice, where—as in humans—experimental autoimmune encephalomyelitis (EAE) severity improves during pregnancy. One of several studies of the pregnancy hormones showed that estradiol and especially estriol delayed EAE onset (11). Another study showed that mice treated with estradiol were protected against EAE (12). This effect was highly dependent on the inhibitory molecule programmed cell death 1 ligand 1 (PDL1 or CD274) since estradiol-treated *Pdl1* knock-out mice lacked protection against EAE. Also, in estradiol-treated mice with intact *Pdl1* decreased levels of pro-inflammatory interleukin (IL)-17, tumor necrosis factor (TNF)-alpha and interferon (IFN)-gamma and an increased level of immunoregulatory IL-10 was observed, while *Pdl1* knock-out mice treated with estradiol showed the reverse pattern (13). Studies of progesterone-treated EAE mice showed reduced neuroinflammation and disease severity with an increase in IL-10 production (14, 15). Also, prolactin is increased during pregnancy, but has shown opposing effects in MS, where both central nervous system tissue protection and stimulation of immune cells have been reported (16). The most beneficial effects of prolactin in EAE was seen in combination with IFN-beta (17). Placental corticotrophin-releasing hormone (CRH), together with the cortisol it stimulates, triggers the production of placental estrogen (18) and during the second trimester of pregnancy, circulating levels of CRH increase exponentially (19). Beneficial effects have been reported of CRH in EAE (20). Likewise, preimplantation factor, produced by the embryo, has anti-inflammatory functions in EAE (21, 22).

In humans a pilot study using oral estriol to treat ten women with MS, six with relapsing-remitting (RR) MS and four with secondary-progressive (SP) MS (23), showed a decrease in IFN-gamma and active magnetic resonance imaging (MRI) lesions in the RRMS group. Also, two clinical studies showed a beneficial effect of estradiol and estriol in women with MS, however with less impact than expected (24, 25).

Our study investigates pregnancy-induced changes in miRNAs and immune cell phenotypes during the 3^rd^ trimester in MS patients. We identified 15 differentially expressed miRNAs compared to postpartum MS patients, confirmed changes in NK-cell subsets and increases in monocytes during pregnancy, and suggested changes in follicular helper T cell subsets. Our data also shows upregulation of IL10 and the co-inhibitory molecules PDL1 and PDL2 during pregnancy, which in monocytes correlated with altered miRNA expression. This, together with bidirectional database predictions of miRNA-target interactions, indicates that these miRNAs and co-inhibitory molecules are involved in the immune regulation observed during pregnancy in MS.

## Materials and Methods

### Study Subjects

We studied 19 pregnant RRMS patients who were recruited as part of the prospective Finnish Multiple Sclerosis and Pregnancy study from neurological centres in Finland and were followed during pregnancy and postpartum. At visits in the outpatient clinic blood samples were drawn and neurological examinations were performed with assessment of relapses and disability determined by the EDSS. A control group of five healthy women was recruited and followed during pregnancy and postpartum, where only blood samples were drawn. Mid-pregnancy (week 26–28) and late pregnancy samples (week 35–37) were representative of the “low disease activity” period whereas postpartum samples taken at week 4–5 and week 10–12 represented the “high disease activity” period. Only Caucasian woman were included in the study. The study was approved by the ethical committee of the Turku University Hospital and written informed consent was obtained from all subjects.

We studied an additional 25 pregnant and 26 non-pregnant untreated RRMS patients, diagnosed with MS according to the 2005 revised McDonald criteria, and recruited from the outpatient clinic at the Danish Multiple Sclerosis Center. All signed the written study informed consent. At visits in the clinic blood samples were drawn, information on relapses was obtained and neurological examinations were performed with assessment of disability as measured by the EDSS. The study was approved by the local Ethics Committee (KF-01 314009).

### miRNA Discovery Study

Total RNA was extracted from peripheral blood mononuclear cells (PBMCs) isolated by Lymphoprep (Axis-Shields, Norway) using the Nucleospin miRNA kit (Merchery-Nagel, Germany) from individuals as presented in **Table 1A** who took part in the prospective Finnish Multiple Sclerosis and Pregnancy study. For detection of miRNA gene expression TaqMan^®^ Array Human MicroRNA Card Set v3.0 containing 754 miRNA targets for detection was used (Life technologies, USA). Quantitative real-time PCR (qPCR) was run on a ViiA7 system and analysis of fold-changes was performed with DataAssist v3.0 using the 2^-ΔΔCT^ method for relative quantification of miRNA levels. Fold changes of miRNA between 3^rd^ trimester and postpartum samples were analyzed with global normalization and miRNAs with p <0.05 were considered differentially expressed between the two periods.

**Table 1:** Clinical characteristics.

A. Clinical characteristics—discovery cohort	MS patients	Healthy individuals	
Female individuals (third-trimester/post-partum)	5/5	5/5	
Age at the beginning of pregnancy, mean (sd)	31.6 (3.2)	27.2 (3.8)	
MS disease duration, mean (sd)	4.7 (3.1)		
EDSS during pregnancy, range, mean (sd)	0–1.5, 0.7 (0.7)		
EDSS at high disease activity time, range, mean (sd)	0–1.5, 0.9 (0.7)		
Number of relapses before pregnancy	2.6 (1.1)		
**B. Clinical characteristics—validation cohort**	**MS patients, third-trimester**	MS patients, post-partum*	**MS patients, untreated**
Female individuals	12	12	12
Age at the beginning of pregnancy or examination, mean (sd)	33.4 (4.0)	30.6 (3.7)	38.0 (11.3)
MS disease duration, mean (sd)	7.9 (5.4)	5.9 (4.4)	7.2 (4.3)
EDSS during pregnancy, range, mean (sd)	0–2.5, 1.1 (0.9)		
EDSS at high disease activity time, range, mean (sd)		0–3.5, 1.2 (1.1)	0–4, 2.2 (1.4)
Number of relapses before pregnancy or 2 years before examination, mean (sd)	3.7 (2.1)**	4.8 (3.5)	1.3 (0.8)

*Post-partum clinical data from one person is missing.

**Relapse data missing from three persons.

### miRNA Validation Study

A validation study of the differentially expressed miRNAs was performed on total RNA. Nucleospin miRNA kit (Machery-Nagel) was used for extraction of RNA from PBMCs collected by Lymphoprep gradient centrifugation. Ten of the twelve 3^rd^ trimester RRMS patients were requited in Denmark, and two were from the prospective Finnish Multiple Sclerosis and Pregnancy study as were the twelve postpartum RRMS patients. All twelve non-pregnant/non-postpartum RRMS patients were requited in Denmark (**Table 1B**). RNA concentration and RNA integrity were measured on a nanochip using the Agilent Bioanalyzer 2100 (Agilent Technologies, CA, USA). Expression of miRNAs was detected by qPCR using pre-amplification and TaqMan-based probes according to manufacturer’s guidelines (**Supplementary Table 1**, Life technologies). In the validation study an expression index was calculated by the 2^-ΔΔCt^ method for relative quantification as previously described (26) using miR-330-3p and miR-196b that were stably expressed miRNAs, as detected in the discovery array study using Normfinder for normalization and a postpartum MS expression index as calibrator.

### Flow Cytometry Analyses of Cell Subpopulations and Immune Activity Markers

Flow cytometry analysis was performed on PBMCs isolated using Lymphoprep and immediately stained with antibody cocktails consisting of fluorochrome-conjugated monoclonal antibodies specific for the surface molecules of monocytes, dendritic cells, B-cells, NK-cells, NKT-cells, CD4+ and CD8+ T-cells and subsets of these cell types, including PDL1 and PDL2, as previously reported (27). In the analysis were twelve unique 3^rd^ trimester pregnant RRMS and 13 non-pregnant RRMS, whereof four were unique, the remaining also included in the expression studies; all were recruited in Denmark (**Table 4A**). Antibodies and isotype controls are listed in **Supplementary Table 2**. Data were collected on a FACS Canto II flow cytometer (BD Biosciences, New Jersey, USA) and analyzed by BD FACS Diva software (BD Biosciences). For data analysis isotype gating controls and visually based criteria were used (**Supplementary Tables 3–5**). Absolute cell counts were calculated using TrueCount tubes (BD Biosciences).

### miRNA and mRNA Gene Expression in Whole Blood Cells

Blood samples were collected in PAXgene tubes (PreAnalytiX, Germany) and RNA was extracted using the PAXgene RNA Blood kit (PreAnalytiX) according to the protocol supplied by the manufacturer, from individuals as presented in **Table 4A**. This was followed by measurement of RNA concentration and integrity on a nanochip as described above. The high capacity cDNA RT-kit (Life Technologies) was used for cDNA synthesis. Following cDNA synthesis, mRNA expression was measured by qPCR analysis using pre-validated TaqMan primer and probe sets (**Supplementary Table 6**) and run on the ViiA7 system (Life Technologies). The expression of *IL10*, *MX1*, *USP18*, *CD274* (encoding PDL1) and *PDCD1LG2* (encoding PDL2) was normalized to the expression of the reference genes *CASC3* and *UBE2D2* and expressed relative to a calibrator sample of pooled cDNA from untreated MS patients (28). Expression index was calculated by the 2^-ΔΔCt^ method using GenEx v.6 Pro (MultiD, Gothenburg, Sweden). To investigate miRNA expression in PAXgene tubes RNA including miRNAs was extracted using the PAXgene miRNA Blood kit (PreAnalytiX) according to manufacturer’s instructions from twelve pregnant MS patients, whereof three are unique and nine are identical with those 3^rd^ trimester patients presented in **Table 1B**. Furthermore, ten non-pregnant MS patients whereof six are unique and four are identical with the RRMS patients in **Table 4B**. The expression of miR-1-3p and miR-625 was normalized to the expression of the reference miRNAs miR-330-3p and miR-196b.

### miRNA and mRNA Expression in Cell Subpopulations

Magnetic separation of monocytes and NK-cells was performed on PBMCs isolated using Lymphoprep. Cells were isolated using MACS cell separation kits (NK Cell Isolation Kit and CD14 MicroBeads) and an autoMACS separator (all from Miltenyi Biotec, Germany). Mean purity of monocytes and NK-cells was 81 and 91%, respectively. Nucleospin miRNA kit (Machery-Nagel) was used for extracting RNA from cell subpopulations (**Table 4B**). All eight 3^rd^-trimester RRMS patients were also in the flowcytometry cohort (**Table 4A**). Two of the non-pregnant MS patients were also in the flowcytometry cohort and four were unique. RNA concentration and integrity were measured on a picochip using the Agilent Bioanalyzer 2100 (Agilent Technologies). A total of 13.8 ng from monocytes and 1.3 ng RNA from NK-cells were used for cDNA synthesis prior to measuring miRNA and mRNA expression. A pre-amplification step was used for miRNAs, and both miRNA and mRNA were detected by qPCR using TaqMan-based probes according to manufacturer’s instructions (Life Technologies). In the study of subpopulations an expression index was calculated by the 2^-ΔΔCt^ method for relative quantification using the reference miRNAs miR-330-3p and miR-196b for normalization and a PBMC pool expression index as calibrator. The expression of *IL10*, *PDL1* and *PDL2* was normalized to the expression of the reference genes *CASC3* and *UBE2D2* (28) and expressed relative to a calibrator sample of pooled PBMC cDNA from untreated MS patients.

### Statistical Analysis

DataAssist v. 3.0 software (Applied Biosystems, USA) and GenEx v.6 was used for the statistical analyses in the discovery screen using a t-test. A p-value of <0.05 was considered statistically significant.

In the validation study a Mann-Whitney U test was used followed by Benjamini-Hochberg correction to correct for multiple testing and false discovery rate q-values of <0.05 were considered statistically significant.

The exploratory analysis of flow cytometry data for possible miRNA targets was investigated by non-parametric analyses using an independent samples Mann-Whitney U test and a p-value <0.05 was considered suggestive and p-value ≤0.005 was considered significant (29).

For miRNA and mRNA gene expression analyses in whole blood and cell subpopulations Mann-Whitney U test was used and a p-value <0.05 was considered statistically significant.

Statistical analyses of flow cytometry and gene expression were performed using GraphPad Prism v.7.

## Results

### Differentially Expressed miRNAs in 3rd Trimester of Pregnancy and Postpartum

The discovery screen of 754 miRNAs in the 3^rd^ trimester compared with the postpartum period was performed in five RRMS patients and five healthy controls (HCs). Clinical characteristics of individuals are presented in **Table 1A**. A total of 232 miRNAs were detected both in HCs and MS patients by qPCR using the TaqMan^®^ Human MicroRNA Array Card Set v3.0. We observed 21 miRNA that were differentially expressed in the 3^rd^ trimester compared to the postpartum period in MS patients (**Table 2**). None of these miRNAs were differentially expressed in HCs (**Supplementary Table 7**). In HCs only one microRNA (miR-18a) was differentially expressed and was 1.3-fold higher in the 3^rd^ trimester than in the postpartum period of pregnancy (data not shown).

**Table 2:** Discovery and validation of differentially expressed miRNAs in third trimester of pregnancy.

Discovery study			Validation study				
Assay ID	MS third-trimester fold change*	MS third-trimester p-value (t-test)	miR ID	MS third-trimester fold change*	p-value (Mann Whitney)	p-value (Benjamini-Hochberg)	Direction fold change
hsa-miR-1-4395333	-12.97	**1.12E-02**	hsa-miR-1	-6.38	**3.70E-04**	**7.50E-04**	down
hsa-miR-17-4395419	-1.80	**2.44E-02**	hsa-miR-17	-1.40	**8.00E-03**	**3.00E-02**	down
hsa-miR-19b-1#-002425	1.83	**2.50E-03**	hsa-miR-19b-1-5p	1.51	**2.00E-02**	**3.00E-02**	up
hsa-miR-20a-4373286	-2.36	**1.40E-02**	hsa-miR-20a	-1.38	**1.20E-02**	8.50E-02	down
hsa-miR-20b-4373263	-1.71	**3.84E-02**	hsa-miR-20b	-1.23	1.00E-01	1.50E-01	down
hsa-miR-22#-002301	3.78	**3.14E-02**	hsa-miR-22*	2.99	**3.33E-05**	**4.90E-04**	up
hsa-miR-28-5p-4373067	-1.94	**1.56E-02**	hsa-miR-28-5p	-1.59	**4.00E-03**	**8.20E-03**	down
hsa-miR-31#-002113	-1.93	**1.44E-02**	hsa-miR-31*	-1.86	**2.00E-02**	**2.10E-02**	down
hsa-miR-95-4373011	-2.85	**3.50E-03**	hsa-miR-95	-2.21	**6.60E-04**	**3.50E-03**	down
hsa-miR-106a-4395280	-1.79	**2.95E-02**	hsa-miR-106a	-1.43	**6.00E-03**	**2.10E-02**	down
hsa-miR-146a-4373132	-1.71	**4.01E-02**	hsa-miR-146a	-1.65	**5.00E-04**	**2.20E-03**	down
hsa-miR-186-4395396	-2.03	**2.22E-02**	hsa-miR-186	-1.41	**1.40E-02**	**3.00E-02**	down
hsa-miR-200b-4395362	-2.00	**2.81E-02**	hsa-miR-200b	-1.18	6.70E-01	3.00E-01	down
hsa-miR-221-4373077	1.61	**9.40E-03**	hsa-miR-221	-1.09	2.90E-01	6.60E-01	up/down
hsa-miR-335-4373045	-2.83	**4.71E-02**	hsa-miR-335	-3.07	**1.41E-05**	**4.20E-04**	down
hsa-miR-342-5p-4395258	-1.74	**4.96E-02**	hsa-miR-342-5p	-1.91	**5.00E-04**	**1.90E-03**	down
hsa-miR-340#-002259	3.12	**1.61E-02**	hsa-miR-340*	2.74	**8.87E-06**	**4.47E-05**	up
hsa-miR-486-5p-4378096	5.39	**2.40E-03**	hsa-miR-486-5p	1.21	8.40E-01	*6.10E-01*	up
hsa-miR-625-4395542	-1.51	**3.85E-02**	hsa-miR-625	-1.71	**1.40E-04**	**7.50E-04**	down
hsa-miR-874-4395379	-2.91	**4.99E-02**	hsa-miR-874	-5.18	**8.88E-06**	**4.90E-04**	down
hsa-miR-922-002152	2.94	**2.76E-02**	hsa-miR-922	-9.80	2.00E-03	2.40E-02	up/down

*Reference: Multiple sclerosis (MS) postpartum. Bold marks fold-change in the same direction as in the discovery screen.

Next, a validation study of the 21 differentially expressed miRNAs was performed in the same extreme periods. Twelve 3^rd^ trimester MS patients and 12 postpartum untreated RRMS patients were investigated (clinical characteristics in **Table 1B**). Sixteen of the 21 miRNAs were validated and were differentially expressed in the same direction as in the discovery screen. After correction for multiple testing 15 miRNA were significant with Benjamini-Hochberg false discovery rates ranging from q = 0.03 to q = 0.000045 (**Table 2**). Of these, 12 miRNAs were down-regulated in the 3^rd^ trimester of pregnancy and 6 of the 15 miRNAs were altered by more than ±2-fold (+2.99- to -6.38-fold **Table 2**) in the same direction as in the discovery screen.

### Differentially Expressed miRNAs in MS Patients During 3rd Trimester of Pregnancy Compared to Untreated Relapsing-Remitting Multiple Sclerosis

Since intravenous immunoglobulin (IVIG) treatment is offered to Danish MS patients from two weeks before birth, we did not have access to untreated postpartum samples for flow cytometry analysis to identify possible miRNA targets of those miRNAs identified in the two extreme states. Thus, we also compared the pregnant RRMS patients with untreated RRMS patients that are in an unrepressed hormonal state and thus are expected to be less extreme than the postpartum patients (**Table 1B**). No significant difference between age, MS disease duration or number of relapses was observed (data not shown). The expression levels of the miRNAs in the untreated RRMS patients were analyzed by qPCR and eight of the validated miRNAs were significantly differentially expressed in the same direction as postpartum samples. After correcting for multiple comparisons two of these miRNAs, miR-1 and miR-625, were still significant, for miR-1 with a more than two-fold difference (**Table 3A**). Comparing postpartum RRMS patients with untreated RRMS patients showed five increased and two decreased significantly changed miRNAs (**Table 3B**). Two miRNAs (miR-22* and miR-340*) showed more than two-fold difference between groups.

**Table 3:** Differentially expresed miRNAs in third trimester of pregnancy and postpartum compared to RRMS.

A. Third-trimester					B. Postpartum			
miR ID	Third-trimester MS fold change*	p-value(Mann Whitney)	p-value(Benjamini-Hochberg)	Direction fold change**	Postpartum MS fold change*	p-value (Mann-Whitney)	p-value(Benjamini-Hochberg)	Direction fold change
hsa-miR-1	-6.41	**6.56E-04**	***8.78E-05***	**down**	1.56	8.85E-01	4.36E-01	up
hsa-miR-17	1.13	7.13E-01	6.50E-01	up	1.37	3.55E-03	**2.82E-02**	up
hsa-miR-19b-1-5p	1.44	**5.00E-03**	2.94E-01	**up**	-1.04	4.70E-01	7.74E-01	down
hsa-miR-20a	-1.08	**1.00E-03**	7.53E-01	**down**	1.22	5.83E-01	1.72E-01	up
hsa-miR-20b	1.13	9.77E-01	6.50E-01	up	1.26	9.99E-02	6.43E-02	up
hsa-miR-22*	-1.00	1.60E-01	9.85E-01	down	-3.53	9.01E-04	**6.50E-04**	down
hsa-miR-28-5p	1.04	3.78E-01	8.73E-01	up	1.46	2.26E-02	**3.46E-02**	up
hsa-miR-31*	-1.01	6.71E-01	9.85E-01	**down**	1.53	5.31E-02	6.43E-02	up
hsa-miR-95	-1.16	**4.50E-02**	6.50E-01	**down**	1.81	3.04E-02	6.43E-02	up
hsa-miR-106a	1.10	1.00E+00	6.50E-01	up	1.36	6.10E-03	**2.82E-02**	up
hsa-miR-146a	-1.30	**6.56E-04**	3.04E-01	**down**	1.21	7.07E-01	1.78E-01	up
hsa-miR-186	-1.12	**6.00E-03**	6.50E-01	**down**	1.22	7.51E-01	1.78E-01	up
hsa-miR-200b	1.24	5.20E-02	3.04E-01	up	1.45	2.14E-01	6.43E-02	up
hsa-miR-221	-1.57	**1.20E-02**	1.68E-01	down	-1.45	1.26E-01	1.58E-01	down
hsa-miR-335	-1.68	**1.00E-03**	2.39E-01	**down**	1.77	1.12E-01	6.43E-02	up
hsa-miR-342-5p	-1.06	5.14E-01	7.53E-01	**down**	1.54	5.11E-03	**2.16E-02**	up
hsa-miR-340*	-1.11	1.78E-01	6.50E-01	down	-5.40	1.23E-04	**1.00E-05**	down
hsa-miR-486-5p	1.50	1.43E-01	4.57E-01	**up**	1.28	3.12E-01	4.88E-01	up
hsa-miR-625	-1.45	**1.44E-04**	***2.58E-03***	**down**	1.15	2.14E-01	1.71E-01	up
hsa-miR-874	1.39	**1.00E-02**	2.27E-01	up	4.17	6.01E-05	6.43E-02	up
hsa-miR-922	3.55	9.32E-01	5.45E-01	up	1.76	1.35E-03	**2.16E-02**	up

Significant changes are marked in bold. * Reference: Untreated relapsing-remitting MS (RRMS). **Bold indicates same direction as Table 2.

### Changes in Immune Cell Subsets and Markers During 3rd Trimester of Pregnancy in Multiple Sclerosis

Due to the very comprehensive list of possible target mRNAs that can be generated from the differentially expressed nominal significant miRNAs, we next analyzed a broad panel of immune cells and activation markers in pregnant MS patients using flow cytometry. The rationale being that immune cell changes could possibly be an effect of altered miRNA expression.

**Figure 1:**
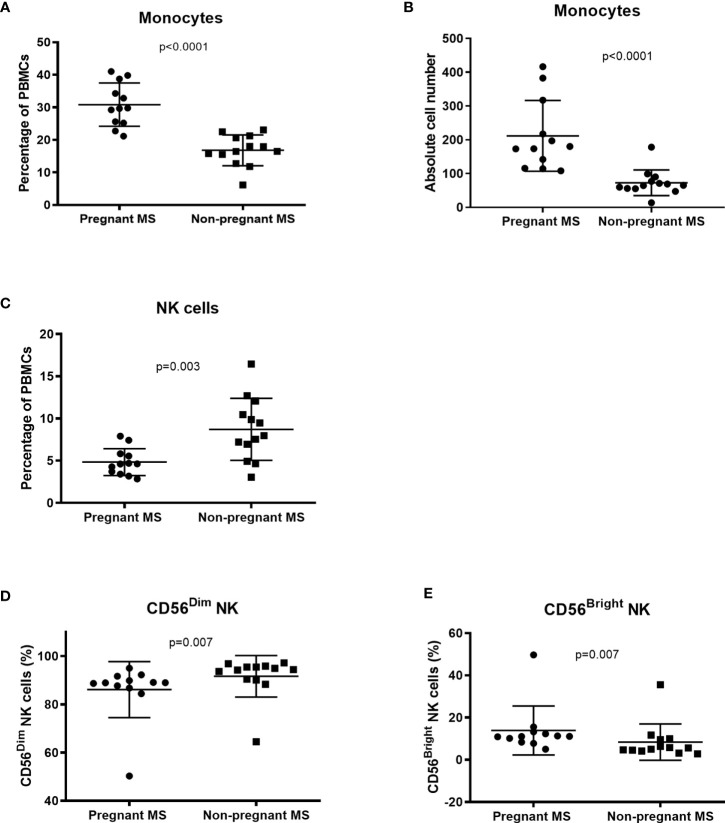
Changes in distribution of cell-subsets during 3rd trimester measured by flowcytometry. Both the percentage **(A)** and absolute number **(B)** of CD14-positive monocytes in PBMC is increased in pregnant MS patients compared to non-pregnant RRMS patients. **(C)** The percentage of NK cells was lower in pregnant MS patients and in this sub-population the percentage of CD56dim was diminished **(D)**, while the percentage of CD56bright was increased in pregnant compared to non-pregnant MS patients **(E)** (Pregnant MS, N = 12, non-pregnant MS, N = 13, boxplot showing mean with SD).

Twelve 3^rd^ trimester pregnant RRMS patients were compared with 13 untreated RRMS patients. Clinical characteristics of the individuals are presented in **Table 4A**. Pregnant MS patients had a highly significant increase in the percentage and absolute cell count of CD14 positive monocytes in blood, compared to non-pregnant RRMS patients (p < 0.0001), respectively, **Figures 1A, B**. In pregnant MS patients a significant decrease was observed in NK-cells compared to non-pregnant MS patients (p = 0.003, **Figure 1C**). A further separation of the cells into CD56^dim/bright^ fractions, showed a lower percentage of CD56^dim^ cells and increased percentage of CD56^bright^ cells in the 3^rd^ trimester of pregnancy (both p = 0.007, **Figures 1D, E**). Expressed in absolute cell counts the CD56^dim^ NK-cells showed a trend to being lower in pregnant MS patients, while the number of CD56^bright^ NK-cells was not altered (p = 0.022 and p = 0.30, respectively, **Supplementary Figures 1A, B**). Furthermore, the percentage of myeloid dendritic cells (mDCs) showed a trend to being lower in pregnant than in non-pregnant MS patients (p = 0.026, **Supplementary Figure 2**). However, mDCs had significantly more surface CCR7 and a tendency of more OX40L and CD11b (ITGAM) (**Supplementary Table 3**). No changes were found in NKT-cells, regulatory T-cells, CD4^+^ and CD8^+^ T -cells, plasmacytoid dendritic cells (pDCs) or B-cells (**Supplementary Tables 3–5**). However, in CD4 T-cells a significantly higher percentage of follicular cells with a non-committed phenotype (CCR6-CXCR3-CXCR5+) was observed in pregnant RRMS patients. In addition, we observed suggestively less CD4 follicular T-cells with a committed Th17.1 phenotype (CCR6+CXCR3+CXCR5+ CD4 T cells, p = 0.035, **Supplementary Table 5**). Otherwise, no significant differences were observed for percentages of investigated immune markers (**Supplementary Tables 3–5**).

**Table 4:** Clinical characteristics.

A. Flowcytometry and qPCR subcohort	Pregnant MS patients	Non-pregnant MS patients
Female RRMS patients	12	13
Age, mean (sd)	33 (3)	34 (8)
MS disease duration, mean (sd)	7.9 (3.4)	5.3 (3.1)
EDSS at examination, mean (sd)	1.2 (1.2)	2.2 (1.7)
MSSS at examination, mean (sd)	1.6 (1.5)	4.2 (3.1)
Delta EDSS previous 2 years, mean (sd)	−0.5 (1)	1 (0.9)
Number relapses previous 2 years, mean	8	18
Years since last relapse (year)	2.9 (2.6)	0.8 (0.6)
Pregnancy week, mean (sd)	36.2 (2.7)	
Number of relapses during pregnancy	0	
**B. Monocyte and qPCR subcohort**	**Pregnant MS patients**	**Non-pregnant MS patients**
Female RRMS patients	8	6
Age, mean (sd)	33 (4)	30 (4)
MS disease duration, mean (sd)	8.3 (4.0)	1.3 (1.5)
EDSS at examination, mean (sd)	1.1 (1.4)	1.7 (1.3)
MSSS at examination, mean (sd)	1.4 (1.8)	3.3 (3.7)
Delta EDSS previous 2 years, mean (sd)	−0.7 (1.0)	0.9 (1.5)*
Number relapses previous 2 years, mean	0.8 (1.0)	2.2 (1.2)
Years since last relapse (years)	2.9 (2.3)	0.5 (0.5)
Pregnancy week, mean (sd)	36.3 (3.2)	
Number of relapses during pregnancy	0	

*Data is missing from two persons.

### Upregulation of Interleukin 10, Programmed Cell Death Ligand 1, Programmed Cell Death Ligand 2, and Downregulation of miR-1 in Whole Blood From Multiple Sclerosis Patients Is Not a Result of Pregnancy-Induced Endogenous Type 1 Interferons

Previously, we reported that IL-10 produced by monocytes may reduce myelin basic protein (MBP)-induced T cell proliferation (30). Thus, we hypothesized that the overall increase in monocytes during pregnancy could be associated with an overall increased expression of *IL10* and that this might be associated with the miRNA expression changes observed in pregnant MS patients. Furthermore, PDL1 has been observed to be correlated with IL10 (31). To analyze this hypothesis, we investigated expression of *IL10*, *CD274*, and *PDCD1LG2* and the two miRNAs significant after correction for multiple testing, miR-1 and miR-625 (**Table 3**), in whole blood in pregnant and untreated MS patients.

**Figure 2:**
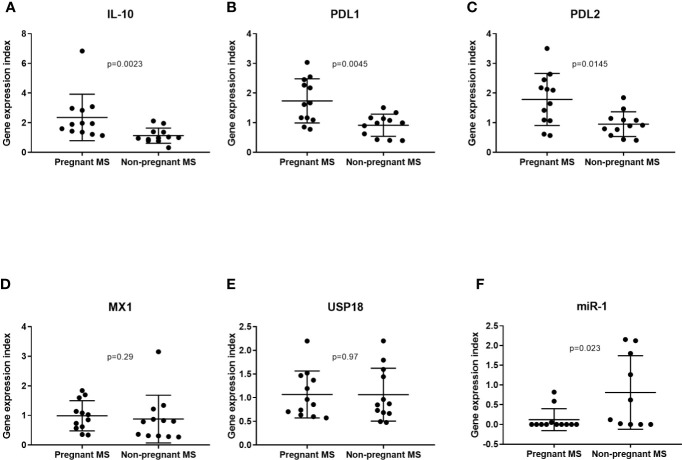
At the whole blood level IL10, PDL1, and PDL2 mRNA were increased in 3rd trimester in MS patients, but not due to an increase in endogenous type 1 interferons during pregnancy. *IL-10, PDL1*, and *PDL2*, respectively **(A–C)**, are found significantly increased in whole blood by real-time qPCR analysis of pregnant MS patients in 3^rd^ trimester compared to untreated MS patients. Gene expression determined by real-time qPCR analysis of the type 1 interferon-induced genes *MX1* and *USP18* **(D, E)** were not significantly changed between pregnant and non-pregnant MS patients. Gene expression of miR-1-3p in whole blood from pregnant MS patients (N = 12) and untreated RRMS patients (N = 10) **(F)** Boxplot showing mean with SD.

*IL10*, *CD274*, and *PDCD1LG2* gene expression were measured by real-time qPCR in individuals presented in **Table 4A**. We observed a significant upregulation of *IL10* and *CD274*, and a trend for *PDCD1LG2* upregulation in pregnant MS patients compared to the untreated RRMS patients (**Figures 2A–C**). Since type 1 interferons (IFN) are known to induce *IL10* and *CD274* (32), we next examined gene expression of the type 1 IFN-induced response genes *MX1* and *USP18*. No difference in the expression levels of these two genes between the pregnant MS patients and untreated patients were observed, suggesting that *IL10* and *PDL1* expression were not induced by endogenous type 1 IFN (**Figures 2D, E**). Comparing whole blood real-time qPCR expression of miR-1 and miR-625 in samples from 3^rd^ trimester of pregnancy to the non-pregnant patients, showed a suggestive downregulation of miR-1, but not miR-625 (result not shown) (p = 0.023, **Figure 2F**). This is consistent with a possible increase in *IL10*, *CD274* and *PDCD1LG2* due to less miR-1 expression during 3^rd^ trimester of pregnancy.

### Gene Expression and Correlation of Differentially Changed mRNAs and miRNAs in the Monocytic Population

**Figure 3:**
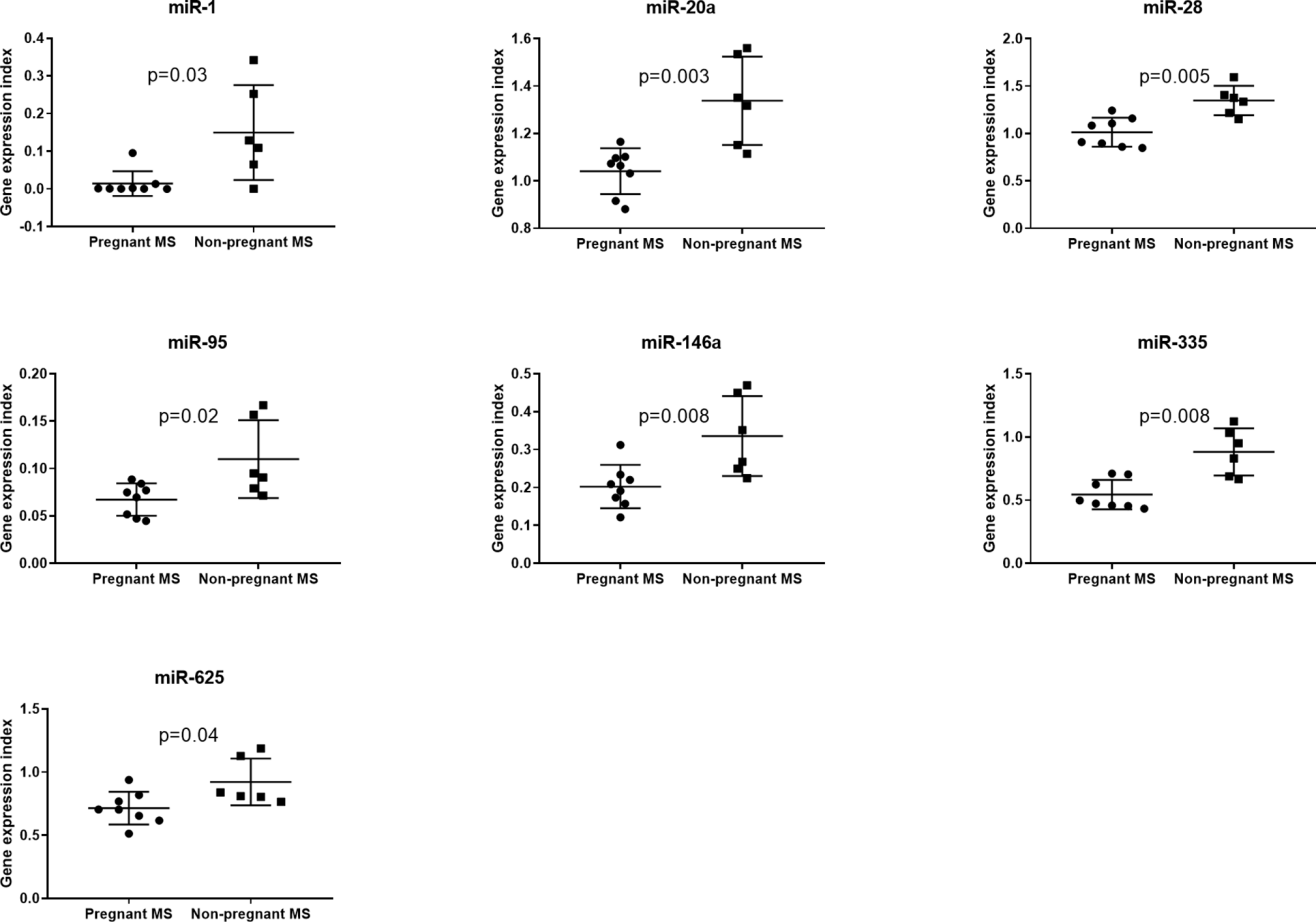
Gene expression of miR-1, miR-20a, miR-28, miR-95, miR-146a, miR-335, and miR-625 was determined by real-time qPCR analysis and found downregulated in monocytes from 3rd trimester pregnant (N = 8) compared with non-pregnant (N = 6) MS patients. No differences were observed in NK-cells from 3rd trimester pregnant MS patients (N = 9) and non-pregnant MS patients (N = 5) (results not shown).

**Figure 4:**
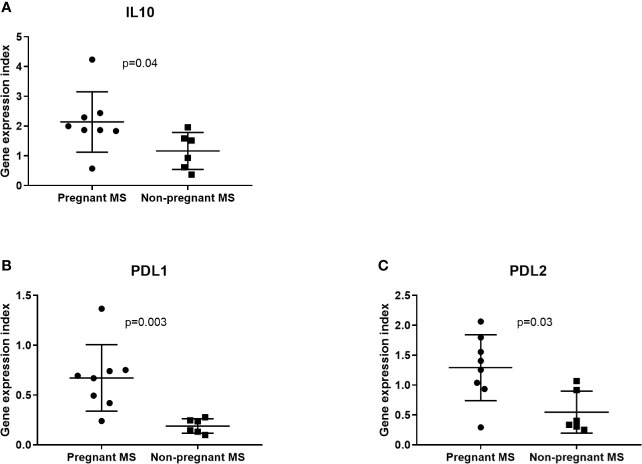
IL10 and immune co-inhibitory marker gene expression determined by real-time qPCR analysis in monocytes from 3rd trimester and untreated non-pregnant MS patients. IL10, PDL1, and PDL2, respectively **(A–C)**, are increased in pregnant (N = 8) compared to non-pregnant (N = 6) MS patients. No differences were observed in NK-cells from 3rd trimester pregnant MS patients (N = 9) and non-pregnant MS patients (N = 5) (results not shown).

Since monocytes and NK-cells were shown to be altered during pregnancy we next investigated in these cell subsets by real-time qPCR analysis the expression levels of the 16 miRNAs that were significantly different between third trimester of pregnancy and post-partum MS patients in the validation study. None of these miRNAs were differentially expressed in NK-cells between pregnant and non-pregnant MS patients (results not shown). In monocytes isolated from eight pregnant MS patients and six non-pregnant MS patients (**Table 4B**) miR-1, miR-95, miR-146a, miR-335, miR-625, miR-20a, and miR-28 were downregulated (p < 0.05, **Figure 3**). Also, the expression of *IL10*, *CD274*, and *PDCD1LG2* was investigated in monocytes and NK-cells. No differences were observed in NK-cells whereas expression of *IL10*, *CD274*, and *PDCD1LG2* was increased in monocytes from pregnant MS patients (p < 0.05, **Figure 4**).

**Figure 5:**
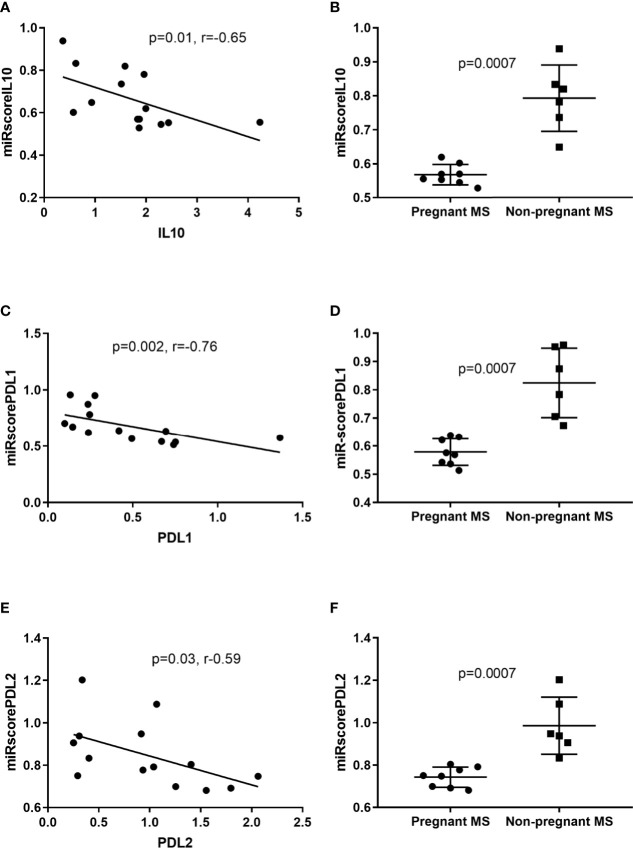
IL10, PDL1, and PDL2 correlation with miRNA scores in monocytes. Negative Spearman correlations were identified between: *IL10* and the miRscoreIL10 computed from miR-1, miR-20a, miR-28, and miR-146a **(A)**. *PDL1* and miRscorePDL1 computed from miR-1, miR-20a, miR-335, and miR-625 **(C)** *PDL2* and miRscorePDL2 computed from miR-20a, miR-28, miR-146a, and miR-625 **(E)**. All miRscores showed a highly significant difference between pregnant and non-pregnant MS patients **(B, D, F)**.

miRNAs either destabilize target mRNA to reduce expression or repress protein translation of its target (10). We therefore conducted a bidirectional search using the database mirDIP (http://ophid.utoronto.ca/mirDIP) to find overlaps between downregulated miRNAs and upregulated mRNAs during pregnancy in monocytes. This database searches in 30 different miRNA target-predicting sources (33). A confidence score was given, where zero assigned the most confident prediction (33). Using “very high confidence” and “high confidence” filters resulted in confidence scores indicating that upregulated mRNAs are targets of the downregulated miRNAs in 3^rd^ trimester pregnant MS patients (**Table 5**). To further elaborate on this, these miRNAs were computed into scores depending on their predicted target from **Table 5**, and correlation between these scores and *IL10*, *CD274*, and *PDCD1LG2* expression, respectively, was investigated. We observed a correlation between *IL10* and the miRscoreIL10 computed from miR-1, miR-20a, miR-28, and miR-146a (p = 0.014, rho = -0.65. **Figure 5A**) and a correlation between *CD274* and miRscorePDL1 computed from miR-1, miR-20a, miR-335, and miR-625 (p = 0.002, r = -0.76. **Figure 5C**) and again a correlation between *PDCD1LG2* and miRscorePDL2 computed from miR-20a, miR-28, miR-146a, and miR-625 (p = 0.03, r = -0.59. **Figure 5E**). All scores showed a significant difference between pregnant and non-pregnant MS patients (p = 0.0007, **Figures 5B, D, F**). When analyzing correlations between *IL10*, *CD274*, and *PDCD1LG2* and single miRNAs, respectively, correlations were seen with those miRNAs marked in bold in **Table 5**.

**Table 5:** Predicted interactions between miRNA and differentially expressed immune markers in monocytes.

Gene Symbol	MicroRNA	Source	Confidence score	Confidence class
IL10	hsa-miR-1-3p	GenMir++, CoMeTa, MAMI	0.113–0.331	Very High
	**hsa-miR-20a-5p**	microrna.org	0.057	High
	**hsa-miR-28-5p**	MultiMiTar	0.094	High
	hsa-miR-146a-5p	CoMeTa	0.106	Very High
PDL1	**hsa-miR-1**	miRTar2GO	0.101	High
	**hsa-miR-20a-5p**	TargetScan	0.266	Very High
	**hsa-miR-20a-5p**	Cupid, DIANA, ElMMo3, miRDB, miRTar2GO, MirAncesTar	0.053–0.101	High
	**hsa-miR-335-5p**	MirAncesTar	0.049	High
	hsa-miR-625-5p	miRTar2GO	0.101	High
PDL2	**hsa-miR-20a-5p**	DIANA, ElMMo3, TargetScan, microrna.org	0.108–0.241	Very High
	**hsa-miR-20a-5p**	CoMeTa, MirTar, Mirza-G, TargetRank, miRDB, miRTar2GO, MirAncesTar	0.070–0.103	High
	hsa-mir-28-5p	microrna.org	0.086	High
	hsa-miR-146a-5p	microrna.org	0.154	Very High
	hsa-miR-625-5p	miRTar2GO	0.101	High

Bidirectional search for targets between downregulated miRNAs and upregulated mRNAs in monocytes between 3rd trimester and untreated MS patients. miRNA marked in bold are suggestively significant negative correlated with its gene target in monocytes (p < 0.05).

### Predicted Associations Between Differentially Expressed Pregnancy miRNA and mRNAs

Next, we considered if the pregnancy-associated changes in miRNAs had any predicted targets among the eleven previously identified genes found to be differentially expressed between 3^rd^ trimester pregnant MS and postpartum MS patients by microarray analysis (7). The miRNAs significantly repressed in the 3^rd^ trimester were used to search for possible interactions with the *TERT*, *PURB*, *CRHR2*, *JUNB*, *IGF2R* genes which are up-regulated during MS pregnancy; this showed that the mRNAs *PURB* and *IGF2R* were possible targets for six and four of the miRNAs, respectively, with the lowest confidence scores seen for *PURB* and miR-20a-5p (**Table 6A**). Also, the opposite scenario was investigated using the miRNAs significantly upregulated in the 3^rd^ trimester to search for their targets in the mRNAs *SOD1*, *NFAT5*, *LSM6*, *GNG11*, *LBR*, and *HNRNPA3* previously identified as downregulated in pregnancy in MS. *LBR* and *NFAT5* were both recognized as possible targets for miR-19b-1-5p, but only in a single data source (**Table 6B**).

**Table 6:** Predicted interactions between differentially expressed miRNAs from this study and differentially expressed genes from previous studies.

6A. Bidirectional search for targets between 3rd trimester downregulated miRNAs from validation study **Table 2** versus pregnancy upregulated genes as identified in Airas et al. (7)				
Gene Symbol	MicroRNA	Source	Confidence score	Confidence class
IGF2R	**hsa-miR-106a-5p**	MultiMiTar	0.109	Very High
	**hsa-miR-146a-5p**	Cupid, TargetScan	0.128–0.147	Very High
	**hsa-miR-17-5p**	MultiMiTar	0.109	Very High
	hsa-miR-20a-5p	MultiMiTar	0.108	Very High
PURB	**hsa-miR-1-3p**	Cupid, TargetScan	0.128–0.150	Very High
	**hsa-miR-106a-5p**	Cupid, ElMMo3, MAMI, MultiMiTar, TargetScan, miRcode	0.125–0.288	Very High
	**hsa-miR-17-5p**	Cupid, ElMMo3, MAMI, MultiMiTar, TargetScan, miRcode	0.114–0.288	Very High
	**hsa-miR-186-5p**	BCmicrO, ElMMo3, TargetScan	0.116–0.288	Very High
	hsa-miR-20a-5p	DIANA, ElMMo3, MAMI, MultiMiTar, TargetScan	0.106–0.288	Very High
	**hsa-miR-335-5p**	MultiMiTar	0.159	Very High
6B. Bidirectional search for targets between 3rd trimester upregulated miRNAs from validation study **Table 2** versus pregnancy downregulated genes as identified in Airas et al. (7)				
**Gene Symbol**	**MicroRNA**	**Source**	**Confidence score**	**Confidence class**
LBR	**hsa-miR-19b-1-5p**	Cupid	0.128	Very High
NFAT5	**hsa-miR-19b-1-5p**	Cupid	0.133	Very High
6C. Bidirectional search for targets between 3rd trimester downregulated miRNAs from validation study **Table 2** versus pregnancy upregulated genes as identified in Gilli et al. (8, 9)				
**Gene Symbol**	**MicroRNA**	**Source**	**Confidence score**	**Confidence class**
CXCR4	**hsa-miR-1-3p**	CoMeTa, TargetRank, TargetScan	0.103–0.237	Very High
	**hsa-miR-106a-5p**	miRcode	0.128	Very High
NR4A2	**hsa-miR-1-3p**	Cupid, ElMMo3, GenMir++, MAMI, PicTar, TargetRank, TargetScan, microrna.org	0.104–0.370	Very High
	**hsa-miR-106a-5p**	Cupid, DIANA, ElMMo3, GenMir++, MAMI, MultiMiTar, TargetRank, TargetScan, microrna.org, miRcode	0.104–0.358	Very High
	**hsa-miR-17-5p**	BCmicrO, Cupid, ElMMo3, GenMir++, MAMI, MultiMiTar, PicTar, TargetRank, TargetScan, microrna.org, miRcode	0.104–0.361	Very High
	**hsa-miR-186-5p**	BCmicrO, Cupid, ElMMo3, TargetRank, TargetScan, microrna.org	0.104–0.288	Very High
	hsa-miR-20a-5p	Cupid, ElMMo3, MAMI, MultiMiTar, PicTar, TargetRank, TargetScan, microrna.org	0.104–0.389	Very High
	**hsa-miR-335-5p**	MultiMiTar	0.106	Very High
	**hsa-miR-95-3p**	TargetRank	0.104	Very High
6D. Bidirectional search for targets between 3rd trimester upregulated miRNAs from validation study **Table 2** versus pregnancy downregulated genes as identified in Gilli et al. (8, 9)				
**Gene Symbol**	**MicroRNA**	**Source**	**Confidence score**	**Confidence class**
FAM49B	**hsa-miR-19b-1-5p**	Cupid	0.128	Very High

Table 6A–D: miRNA marked in bold are significantly differentially expressed after Benjamini–Hochberg correction as shown in the validation study (**Table 2**).

In another study of gene expression in samples from pregnant MS patients a signature of seven genes was found to be differentially expressed at the mRNA level when comparing MS patients and HCs before, during and after pregnancy (8). Additionally, three genes from this signature, *SOCS2*, *NR4A2* and *TNFAIP3* were found to be lower expressed in MS patients with an aggressive disease course, indicating that they might function as negative regulators of inflammation (9). Thus, we searched mirDIP to address whether the downregulated 3^rd^ trimester miRNAs we identified have the MS pregnancy-upregulated genes *SOCS2*, *NR4A2*, *TNFAIP3*, and *CXCR4* as predicted targets. Only two mRNAs were targets with the applied settings. *CXCR4* was target for two miRNAs, whereas *NR4A2* was target for seven of the miRNAs in many different sources with a confidence score ranging from 0.104–0.389 (**Table 6C**). Also, we searched for predicted targets of the 3^rd^ trimester up-regulated miRNAs identified in this study in the 3^rd^ trimester downregulated mRNAs *POLR2J*, *FAM49B*, *STAG3L1* from the seven-genes-signature (8). Here, only *FAM49B* was recognized as possible target of miR-19b-1-5p in a single source but with a low confidence score (**Table 6D**). Thus, at high stringency settings, nine of the 15 differentially expressed miRNAs had a predicted target in one or more of the mRNAs previously identified to be regulated during pregnancy.

## Discussion

The major aim of this study was to analyze miRNA levels and immune cell phenotypes during the 3^rd^ trimester of pregnancy in MS patients where disease activity is ameliorated. We first profiled the expression of miRNAs in a discovery cohort where we compared miRNAs among MS patients in 3^rd^ trimester compared to postpartum where disease activity is aggravated. We validated these changes by comparing 3^rd^ trimester to both postpartum and untreated MS patients. The changes were confirmed although changes were more pronounced for 3^rd^ trimester and postpartum patients. Secondly, in pregnant MS patients, we found increased levels of monocytes and found that their increased gene expression of *IL10*, *CD274* (PDL1) and *PDCD1LG2* (PDL2) mRNA contrasted to the reduced expression of seven miRNAs in this cell type, possibility indicating absence of translational repression or mRNA destabilization by miRNAs. Third, the increase of *PDL1*, *PDL2*, and *IL10* mRNA in whole blood was found not to be a result of potentially pregnancy-induced endogenous type 1 interferons. Fourth, we confirmed modulation of circulating NK-cells, represented by fewer CD56^dim^ and more CD56^bright^ NK-cells, and observed an increase in non-committed follicular helper T cells. Finally, we explored the regulatory potential of the identified miRNAs in previously and herein reported differentially expressed mRNAs during pregnancy and found negative correlations between miRNAs and their putative targets.

Among the 15 miRNAs differentially expressed during the 3^rd^ trimester period of pregnancy, where disease activity is most attenuated, most were downregulated compared to postpartum: miR-1, miR-17, miR-28-5p, miR-31*, miR-106a, miR-146a, miR-186, miR-335, miR-342-5p, miR-625, and miR-874. Of these miR-1, miR-17, and miR-146a were previously reported to be upregulated in MS patients in PBMCs (miR-146a) (34–36), in CD4+ T cells (miR-1, miR-17) (36) or EAE-susceptible rats (miR-146a, miR-1) (37), implying an immunomodulatory effect of these miRNAs in MS. Furthermore, estrogen-treated mice had downregulated miR-146a in splenic lymphocytes (38) and not only in MS, but also in RA, miR-146 dysregulation has been reported (39).

The miRNAs miR-17, miR-20a, and miR-106a downregulated in the 3^rd^ trimester are part of the miR-17-92 and miR-106-363 clusters and are associated with proliferative functions (40). Knock-out of the miR-17-92 cluster in EAE mice ameliorated symptoms compared to wildtype, in agreement with the assumption that downregulation of these miRNAs in 3^rd^ trimester can reduce disease activity. miR-19b, also contained in the miR-17-92 cluster, was one of the three upregulated miRNAs (miR-19b-1-5p, miR-22*, and miR-340*) in PBMCs during pregnancy, the miR-17-92 miRNAs are known to be individually regulated at the post-transcriptional level and have different mRNA targets (40).

The 3^rd^ trimester-downregulated miR-28 in PBMCs has also been associated with proliferative functions. This microRNA was upregulated and increased cell proliferation in gastric cancer, possible by repressing PTEN that is a repressor of PI3K/AKT signalling (41).

In the discovery study none of the significantly regulated miRNAs in MS patients were significantly differently expressed in HCs, however the direction of miRNA fold-changes were the same, strengthening our findings. This was especially true for the miRNAs; miR-1, miR-28, miR-95, miR-146a, miR-186, and miR-625, implying that the effects of pregnancy hormones might have a stronger regulatory effect on these miRNAs in MS patients than in healthy individuals. This could be due to differences in environmental and genetic factors and a pathologically skewed immune system that could act synergistically on regulation. However, it could also be explained by the small size of the discovery cohort.

When comparing the 15 differentially expressed miRNAs with non-hormonal-repressed untreated MS patients instead of postpartum MS patients, eight miRNAs were still significantly differentially regulated in PBMCs, including the above-named miRNAs, except for miR-28. This supports these miRNAs’ regulation by pregnancy hormones, with miR-1 and miR-625 changes still significant after correction for multiple comparisons.

The most significantly decreased miRNAs in the postpartum stage, compared with untreated RRMS patients, were miR-22* and miR-340*; these miRNAs could potentially be involved in MS disease exacerbations. Since postpartum samples were not available from the Danish outpatients clinic, flow cytometry analysis was not possible and identification of potential targets of these miRNAs were not studied further.

We showed that monocyte counts are increased in 3^rd^ trimester compared to untreated MS patients. While this is a new observation in MS patients, a previous study reported an increase of monocyte gene expression signatures during pregnancy in persons with RA and HCs (42), indicating that this could be a general effect of pregnancy.

In a pilot study, Soldan et al. found a decrease in CD4 and CD8 T cells and an increase in B cells while CD64 positive monocytes/macrophages were unchanged after estriol treatment (43). We did not observe changes in these PBMC subsets. This discrepancy might in part be explained by the estriol treatment levels being comparable to only 6 months of pregnancy and the fact that estriol is only one of many pregnancy factors that could be responsible for lowering disease activity during pregnancy. Furthermore, methodically we used very different setups.

We confirmed a relative increase in CD56^bright^ NK-cells in pregnant MS patients, which is in accordance with a regulatory potential of this NK-cell subset. Interestingly, this subset is also increased by treatment with daclizumab and interferon-beta, and MS patients responding well to fingolimod treatment were recently shown to have more CD56^bright^ NK-cells (44).

PDL1 and PDL2 are ligands of the programmed death receptor 1 (PD-1) and upon binding to this receptor a co-inhibitory signal can reduce T cell proliferation. The immunoregulatory potential of co-stimulatory/inhibitory molecules depends on their balanced signalling. This balance appears to be weighted toward more inhibitory molecules during pregnancy in MS patients in the present study, as we find increased expression of *CD274* (PDL1) and (*PDCD1LG2*) PDL2in monocytes. This increase was not shown at the protein level, possibly explained by the dominance of miRNA mediated mRNA destabilization compared to translational repression. Previously, pathways involving these molecules were implicated in MS (45). In stable MS patients, higher levels of PDL1 and IL10 were observed in monocytes and B cells after MBP stimulation compared with active MS patients (46) and downregulation of immunosuppressive molecules PD-1 and PDL1 was observed in MS patients compared with healthy individuals (47). These studies did not report of changes in PDL2 in MS. We did not find major differences in T cell subsets in the present study, but we did find an increase in non-committed follicular CD4 T cells during pregnancy which should be studied in more detail in future studies.

We find it interesting that pregnant RRMS patients had more CCR7 positive mDCs in the blood, since a recent finding in mice showed CCR7 positive dendritic cells used CCR7 to escape the CNS and the retention of CCR7 negative dendritic cells was associated with exacerbation of EAE (48). Whether a similar mechanism could be operating in pregnant patients with MS remains to be established.

In mice the protective effect of estrogen was mediated by B cells expressing PDL1, whereas we observed PDL1 to be expressed on monocytes and dendritic cells rather than B cells as assessed by flow cytometry (13).

Our finding that MS patients had an overall increase in both monocyte number, *IL10*, *CD274* and *PDCD1LG2* during the 3^rd^ trimester led us to speculate that endogenous type 1 interferons could be induced during pregnancy, since previous results have shown increased PDL1 and *IL10* expression in monocytes after IFN-beta treatment (49), resulting in a strong anti-proliferative effect on CD4 T cells (32). Such effects might explain some of the amelioration in relapse activity during 3^rd^ trimester of pregnancy, however our study clearly ruled out that *PDL1* and *IL10* were increased due to changes in type 1 interferons, since the type 1 interferon-induced genes, *MX1* and *USP18*, were not differentially expressed in pregnant and non-pregnant women with MS.

The most consistent miRNA change in the present study was observed for miR-1 that showed more than six-fold downregulation in PBMCs in 3^rd^ trimester compared to both postpartum and untreated RRMS patients. At the whole blood level and in monocytes, we also found downregulation of miR-1 in pregnant MS patients compared with non-pregnant MS patients, however with less fold differences. Paradoxically, this miRNA is considered a tumor suppressor gene and its lower expression in tumor cells is coupled to cell proliferation *via* upregulation of Notch2 (50). Downregulation of miR-1 was also observed in CD14 positive monocytes in patients with acute myocardial infarction, at a stage with increased monocyte levels (51). Importantly, miR-1 seem to be important in cardiac regulation, and heart failure was observed in miR-1 knock-out mice making this miRNA less attractive for future anti-miRNA-treatment (52).

We found none of the 15 differentially expressed miRNAs in PBMCs to be differentially expressed in NK-cells between pregnant and non-pregnant MS patients. This may be influenced by the very low RNA input from NK-cells; thus, we cannot rule out effects of pregnancy-regulated miRNAs in this cell subset. A ten-fold higher RNA input was used from monocytes where differential miRNA expression between pregnant MS and untreated MS patients was readily observed. Some contamination of miRNAs from other cell subsets could also be expected, since their purity was not optimal.

Since miRNAs can act synergistically it is an interesting observation that a strong negative correlation exists between miRNAs targeting *CD274* (PDL1) and its gene expression in monocytes. Also, *IL10* and *PDCD1LG2* (PDL2) targeting miRNAs showed a tendency to be negative correlated with *IL10* and *PDCD1LG2* gene expression in monocytes. Furthermore, the expression of these miRNA scores and mRNAs were significantly different between pregnant MS patients and untreated MS patients, where disease activity is on average lower compared with the first few months postpartum. This suggests a regulatory impact of these miRNAs in monocytes that may, at least partly, mediate the disease-ameliorating effect of pregnancy.

miR-146a was also downregulated in 3^rd^ trimester monocytes. Results from mirDIP database predicted that miR-146a has *IL10* and *PDCD1LG2* as targets with very high confidence. A recent study found that miR-146a was increased in the corpus callosum in the cuprizone mice model of MS and that miR-146 knock-out mice had reduced demyelination and axonal loss (53). Accordingly, we found in high stringency mirDIP searches the differentially expressed miRNAs to have predicted targets among previously reported mRNA targets changed during pregnancy. Thus, the pregnancy-induced miRNAs might also regulate these mRNA targets in the blood compartment. However, we did not measure these mRNAs and their correlation and functional impact would have to be further validated.

We recognize that the use of small cohort sizes may limit the extent to which our results can be generalized. The use of combined sub-cohorts reflects that recruitment of these patients was complex due to both their circumstance and the analyses performed. Also, due to the variability of the disease course in MS this can be critical when investigating miRNA and their complex regulatory networks, but at least during pregnancy the strong hormonal influence was present in all women, and RRMS patients were all untreated. We are aware that direct correlations between miRNAs or immunologically active molecules and clinical or MRI measures of disease activity are needed to formally prove the functional impact of these changes. However, the wide variety of treatments regimens used before pregnancy rendered such correlations unreliable. Also, postpartum samples for further investigation beyond the discovery and validation study would have strengthened our analyses of this disease exacerbating stadium, but the wide use of intravenous immunoglobulin therapy postpartum in Denmark precluded this option.

To summarize we have shown differential expression of miRNAs in the blood in the 3^rd^ trimester of pregnancy and postulate that some of these may mediate the beneficial effect of pregnancy; however this study is mainly descriptive, and more studies with functional assays in blood cells are needed before considering the possible use of miRNA inhibitors *in vivo* in order to mimic the effects of miRNAs downregulated during pregnancy (54).

## Data Availability Statement

The study data are unsuitable for public deposition due to ethical restrictions and privacy of participant data. Data are available for any interested researcher who meets the criteria for access to confidential data. The corresponding author may be contacted to request study data.

## Ethics Statement

The study was approved by the ethical committee of the Turku University Hospital and by the Danish local Ethics Committee (KF-01 314009). Written informed consent was obtained from all subjects.

## Author Contributions

HBS and FS conceived and designed the study. HBS wrote the first draft of the manuscript and prepared the figures and tables. LA, JRC, LB, and AO were responsible for the patient sample and data collection. HBS and FS were responsible for the gene expression analyses. HBS, JRC, BRN, LB, and FS were responsible for flow cytometry analyses. All authors contributed to the article and approved the submitted version.

## Funding

This research was supported by grants from the Danish Council for Strategic Research, the Danish Multiple Sclerosis Society, Emil C. Hertz and Inger Hertz’ Foundation, the Johnsen Foundation, and the Warwara Larsen Foundation.

## Conflict of Interest

LA has received institutional research funding from Biogen, Novartis, Roche, and Merck. She has obtained congress and travel fees from Sanofi Genzyme, Teva, Merck, Biogen, Roche, Novartis, and compensation for lectures and advising from Novartis, Sanofi Genzyme, Teva, Merck, Biogen, and Roche. AO has served on scientific advisory boards for Biogen and Genzyme; has received support for congress participation from Biogen, Novartis, Genzyme, and TEVA; has received speaker honoraria from, Biogen, Novartis, and TEVA. FS has served on scientific advisory boards, been on the steering committees of clinical trials, served as a consultant, received support for congress participation, received speaker honoraria, or received research support for his laboratory from Biogen, EMD Serono, Merck, Novartis, Roche, Sanofi Genzyme, and Teva.

The remaining authors declare that the research was conducted in the absence of any commercial or financial relationships that could be construed as a potential conflict of interest.
