# Supplementary figures and images for: Pregnancy-Induced Changes in microRNA Expression in Multiple Sclerosis
Source: Front Immunol. 2021 Jan 28;11:552101. doi: 10.3389/fimmu.2020.552101 (PMC7876450; doi:10.3389/fimmu.2020.552101)

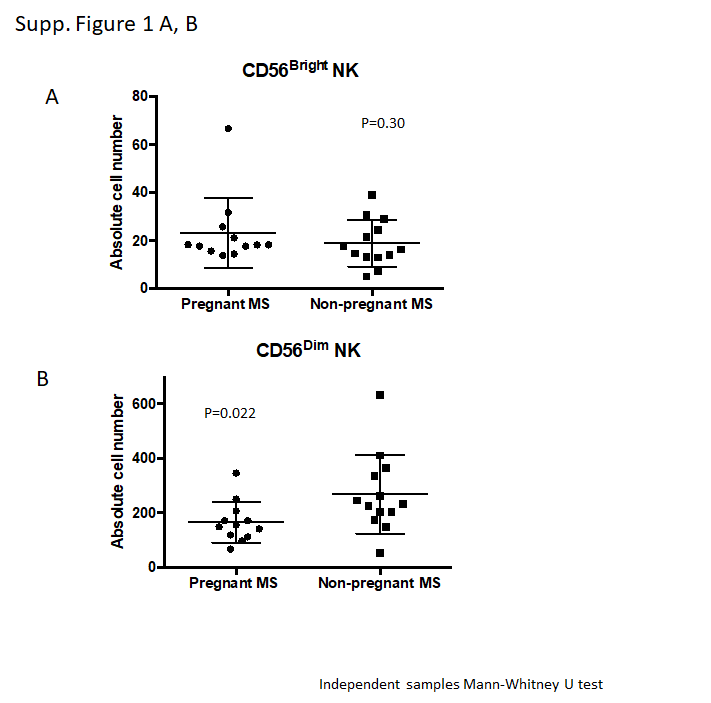

Supplement: Supplementary file 1 [file Image_1.tif]

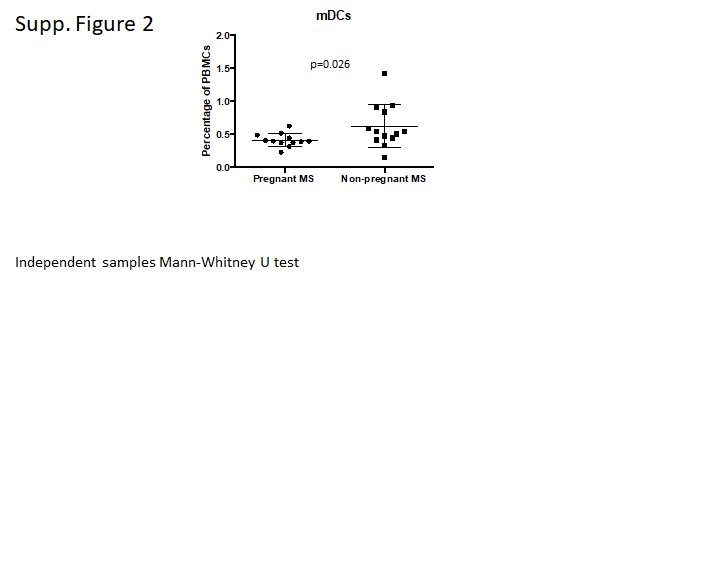

Supplement: Supplementary file 2 [file Image_2.tif]
